# Bridging the gap: improving liaison psychiatry documentation quality to meet the National Confidential Enquiry into Patient Outcome and Death (NCEPOD) treat as one recommendations at newcastle hospitals

**DOI:** 10.1192/bjo.2021.567

**Published:** 2021-06-18

**Authors:** David Ou, Sara Ibrahem, Sahar Basirat, Sarah Brown

**Affiliations:** 1 The Newcastle upon Tyne Hospitals NHS Foundation Trust; 2 Cumbria, Northumberland, Tyne And Wear NHS Foundation Trust

## Abstract

**Aims:**

This project aimed to assess and improve the quality and frequency of documentation from Psychiatric Liaison Team (PLT) to ward-based medical colleagues against the Treat as One recommendations. From experience, we hypothesised that written documentation of information crucial to patient care is not consistently meeting standards. This communication breakdown directly affects patient safety, potentially introducing additional risks to our already vulnerable patient group.

Effective communication between PLT and our medical colleagues bridges the gap in providing continuity of care and ensures patients’ mental and physical health needs are met in acute trusts. The NCEPOD found that there remains many barriers to high quality mental healthcare provided to patients in general hospitals and recommended 7 elements that PLT documentations should encompass.

**Method:**

We audited initial PLT assessments and the resulting documentation to determine if these met the 7 standards set by NCEPOD. Baseline audit undertaken from 21-27/09/2020 encompassing 130 patient referrals to PLT.

A period of time was allotted to implement robust changes to improve the service. This included a streamlined e-template that automatically populates in the acute hospital eRecord system which prompts clinicians to document according to the NCEPOD standards, structured clinician training and education, and the nomination of “Treat as One Guardians” in the team to ensure that acute trust documentations are present during daily multidisciplinary meetings.

The cycle was then completed on 22-28/02/2021 with a re-audit capturing 55 referrals.

**Result:**

Implementation of our recommended changes saw an increase from 58% of documentations with ≥50% NCEPOD elements to 98% in the re-audit.

We also saw an increase in number of the NCEPOD 7 elements included following intervention: formulation (0% to 8%), legal status and capacity (47% to 79%), risk assessment (2% to 28%), risk management (18% to 53%), and discharge plan (2% to 29%).

Completion rate of acute trust documentation increased from 74% to 96%.

Our interventions also led to more contemporaneous communication, significantly reducing mean time from assessment to documentation in both acute trust and mental health records from 6.02 to 3.53 hours, (p = 0.04) and 6.12 to 3.50 hours, (p = 0.05) respectively.

**Conclusion:**

Following our interventions, the results showed improving trends in the frequency and quality of our documentation with secondary outcomes showing increased documenting efficiency. Our current practice is not yet optimal and retains potential to adversely affect our patients. We propose further investigating barriers to change using the quality improvement PDSA (Plan, Do, Study, Act) methodology to continue innovating.

